# The Detection of Mutations and Genotyping of Drug-Resistant *Mycobacterium tuberculosis* Strains Isolated from Patients in the Rural Eastern Cape Province

**DOI:** 10.3390/idr15040041

**Published:** 2023-07-10

**Authors:** Lindiwe M. Faye, Mojisola C. Hosu, Selien Oostvogels, Anzaan Dippenaar, Robin M. Warren, Ncomeka Sineke, Sandeep Vasaikar, Teke Apalata

**Affiliations:** 1 Department of Laboratory Medicine and Pathology, Walter Sisulu University, Mthatha 5099, South Africa; mojisolahosu@gmail.com (M.C.H.); ncomekasineke3@gmail.com (N.S.); sandeepvasaikar@yahoo.com (S.V.); ruffinapalata@gmail.com (T.A.); 2 National Health Laboratory Services (NHLS), Mthatha 5099, South Africa; 3 Family Medicine and Population Health (FAMPOP), Faculty of Medicine and Health Sciences, University of Antwerp, BE-2000 Antwerp, Belgium; selienoostvogels@hotmail.com (S.O.); anzaan.dippenaar@uantwerpen.be (A.D.); 4 DSI-NRF Centre of Excellence for Biomedical Tuberculosis Research, South African Medical Research Council, Parowvallei, Cape Town 7505, South Africa; rw1@sun.ac.za; 5 Centre for Molecular and Cellular Biology, Division of Molecular Biology and Human Genetics, Faculty of Medicine and Health Sciences, Stellenbosch University, Cape Town 7505, South Africa

**Keywords:** drug-resistant TB, heteroresistance, mutations, spoligotyping

## Abstract

Drug-resistant tuberculosis (DR-TB) is still a major public health concern in South Africa. Mutations in *M. tuberculosis* can cause varying levels of phenotypic resistance to anti-TB medications. There have been no prior studies on gene mutations and the genotyping of DR-TB in the rural Eastern Cape Province; hence, we aimed to identify DR-TB mutations, genetic diversity, and allocated lineages among patients in this area. Using Xpert^®^ MTB/RIF, we assessed the rifampin resistance of sputum samples collected from 1157 patients suspected of having tuberculosis. GenoType MTBDR plus VER 2.0 was used for the detection of mutations causing resistance to anti-TB medications. The next step was to spoligotype 441 isolates. The most prevalent rifampin resistance-conferring mutations were in *rpoB* codon S531L in INH-resistant strains; the *katG* gene at codon S315TB and the *inhA* gene at codon C-15TB had the most mutations; 54.5% and 24.7%, respectively. In addition, 24.6% of strains showed mutations in both the *rpoB* and *inhA* genes, while 69.9% of strains showed mutations in both the *katG* and *rpoB* genes. Heteroresistance was seen in 17.9% of all cases in the study. According to spoligotyping analysis, Beijing families predominated. Investigation of the evolutionary lineages of *M. tuberculosis* isolates can be carried out using the information provided by the study’s diversity of mutations. In locations wherein these mutations have been discovered, decision-making regarding the standardization of treatment regimens or individualized treatment may be aided by the detection frequency of *rpoB*, *katG*, and *inhA* mutations in various study areas.

## 1. Introduction

Global public health continues to be threatened by tuberculosis (TB), an infectious disease caused by *Mycobacterium tuberculosis* (*M. tuberculosis*) and ranked among the top 10 causes of mortality globally [1]. In 2020, the estimated number of incident cases of TB stood at 9.9 million, with 25% of cases occurring in Africa. South Africa is one of the 30 countries with the highest TB burden, and has the eighth highest TB incidence globally, with more than 500 cases per 100,000 population. This towers above the global average incidence of 127 cases per 100,000 population [2].

In South Africa, TB continues to be a disease of major importance, and it remained the leading cause of death for three consecutive years from 2016 to 2018 [3]. In 2019 alone, an estimated 360,000 South Africans became ill with TB, and 58,000 people were estimated to have died from the disease [2]. The COVID-19 pandemic’s effects have undone years of progress made in reducing the number of TB deaths worldwide, with the predicted number of deaths in 2020 returning to the level of 2017 [4]. The eradication of TB by 2035, a strategic goal of World Health Organization (WHO), cannot be actualized unless the emergence of resistance in TB is seriously addressed and controlled [5].

Drug-resistant TB (DR-TB) is TB caused by *M. tuberculosis* that is resistant to TB medications. Different types of DR-TB are drug monoresistant, multi-drug-resistant (MDR), pre-extensively drug-resistant (Pre-XDR), and extensively drug-resistant (XDR). In this study, mono drug-resistant TB is defined as *M. tuberculosis* that is resistant to either isoniazid (INH) or rifampicin (RIF). Both MDR-TB (defined as TB that is resistant to at least INH and RIF) and XDR-TB (defined as MDR-TB plus resistance to any fluoroquinolone and at least one second-line injectable drug) have emerged as serious public health issues, and present new challenges for international TB control efforts [6]. Treating MDR-TB and XDR-TB takes longer than treating drug-susceptible TB, has severe side effects, and frequently has poor treatment outcomes [6,7]. Drug-resistant TB (DR-TB) has emerged as a major risk to global TB control. Different mutations in genes such as *rpoB*, *katG*, *inhA*, *pncA*, *embB*, *rpsL, gyrA*, *ethA* and *rrs* have been identified as conferring resistance to TB first-line drugs, second-line drugs or injectables, and fluoroquinolones [8,9]. Mutations in the codon 507 to 533 regions of the *rpoB* gene, called the rifampicin resistance-determining region (RRDR), are mainly responsible for rifampicin resistance, while mutations in the *katG* and *inhA* genes are associated with INH resistance [9]. *katG* and *inhA* mutations give rise to high-level and low-level INH resistance, respectively [8,9]. Although mutations in both *gyrA* and *gyrB* genes are responsible for fluoroquinolone resistance, *gyrA* accounts for 60–70% of all mutations [10,11,12]. Recent research has revealed that different mutations in *M. tuberculosis* can confer varying levels of phenotypic resistance to anti-TB medications [8,9,13]. Consequently, the aggregation of mutations at several positions has a comprehensive effect on drug resistance [14].

Heteroresistance occurs when a patient contracts both resistant and susceptible strains of an infection at the same time, or when an antibiotic-resistant genetic change occurs in a single clone [15]. Heteroresistance is viewed as a precursor to full resistance or low levels of drug-resistant TB [16]. The rate of false-negative drug resistance results detected by phenotypic susceptibility tests rises when heteroresistance is linked to mixed infections of *M. tuberculosis*. [17,18,19]. Therefore, heteroresistance, which is particularly difficult to detect with a phenotypic technique in the initial stage, might interfere with both the diagnosis and the therapy. In areas with high rates of tuberculosis, particularly MDR-TB, mixed infections and heteroresistance may be particularly prevalent [20], and they can promote the spread of drug-resistant strains of *M. tuberculosis*, accelerate the pace of treatment failure, and aid the strains in acquiring mutations. A mixed infection, defined as the presence of strains with different patterns in two or more loci, is one of the most frequent mechanisms that explains heteroresistance. Other mechanisms include clonal heterogeneity, which results from the division of a single lineage into susceptible and resistant clones as part of its biological evolution [16]. The goal of the research was to determine DR and gene mutations, which continue to be significant obstacles to successful TB control and management in rural parts of the Eastern Cape. There is no information on the prevalence of gene alterations linked to resistance to the antibiotics rifampicin (RIF) and isoniazid (INH) in the context of this investigation. Hence, in this study, we report on the prevalence of mutations in the drug-resistant genes *rpoB*, *katG*, and *inhA*, and identify the strains and lineages of DR-TB strains.

## 2. Materials and Methods

### 2.1. Study Site, Patient Population, and Specimen Collection

Sputum specimens were obtained from 1157 TB suspect patients in different healthcare facilities and sent for testing and analysis to the National Health Laboratory Services (NHLS) in Mthatha over a 36-month period (January 2018 to December 2020). These specimens were collected from patients showing clinical signs of TB by clinicians in various healthcare facilities. The patients were TB suspects and seeking medical help from 118 healthcare facilities located in five districts: Oliver Reginald (OR) Tambo, Alfred Nzo, Amathole, Chris Hani, and Joe Gqabi.

### 2.2. Laboratory Analysis

Three procedures were used to analyse samples, as follows.

#### 2.2.1. Xpert^®^ MTB/RIF Assay

This technique was used to find mutations in the RRDR of *M. tuberculosis* directly on TB sputum samples. The sputum samples were first decontaminated, then a 2:1 reagent buffer containing NaOH and isopropanol was added, followed by 15 min of incubation at room temperature [21]. The Xpert MTB/RIF cartridge containing 2 mL of the final sample was then put into the GeneXpert equipment, wherein the testing procedure is totally automated. The experiment resulted in samples being categorised as RIF-resistant or susceptible, and *M. tuberculosis* negative or positive.

#### 2.2.2. Phenotypic Drug Susceptibility Testing (DST)

According to the manufacturer’s instructions, the test was performed on sputum samples using an automated BACTEC Mycobacterial Growth Indicator Tube (MGIT) 960 (Becton Dickinson, Franklin Lakes, NJ, USA). Isoniazid (0.1 g/mL of medium), rifampin (1.0 g/mL of medium), ofloxacin (2.0 g/mL), amikacin (1.0 g/mL), kanamycin (2.5 g/mL), and capreomycin (2.5 g/mL) were used at their critical concentrations for the first- and second-line anti-TB medications [6,7].

Briefly, reconstitution of PANTA powder with growth supplement (15 mL) was carried out, followed by its insertion into the MGIT tube and the inoculation of each tube with 0.5 mL of the processed specimen. The tubes were incubated at 37 °C in the BACTEC MGIT 960 instrument and checked automatically every 60 min for increased fluorescence. Culture tubes were maintained until they became positive, or for a maximum of 42 days to confirm if negative. Positive samples were removed from the instrument and recorded as positive along with the number of incubation days.

#### 2.2.3. Genotypic DST

In this study, a deoxyribonucleic acid (DNA) strip-based method called GenoType MTBDR plus version 2.0 (Hain Lifescience, Nehren, Germany) was used to simultaneously detect the most significant *rpoB* mutations, which confer RIF resistance, and *katG* and *inhA* mutations, which confer high- and low-level INH resistance, respectively [6,7]. Following the manufacturer’s instructions, three procedures—DNA extraction, multiplex amplification using biotinylated primers, and reverse hybridisation—were carried out [22].

DNA extraction was performed using a GenoLyse kit (Hain Lifescience, Nehren, Germany). Briefly, 1 mL of the decontaminated sputum was centrifuged for 15 min at 10,000× *g*, and the supernatant was discarded. The sample was incubated at 95 °C for five minutes after the addition of 100 μL of lysis buffer in order to re-suspend the sediment. Subsequently, 100 μL of neutralisation buffer was added, vortexed, and centrifuged for five minutes [23]. Multiplex amplification was carried out in different cycles at different temperatures as follows: 1 cycle of 15 min at 95 °C, 20 cycles of 30 s each at 95 °C and 2 min at 65 °C, 30 cycles of 25 s each at 95 °C, 40 s at 50 °C, and 40 s at 70 °C, and finally, 1 cycle of 8 min at 70 °C. Reverse hybridisation was performed using an automated hybridisation system, Auto-Lipa 48, (Innogenetics), following the manufacturer’s instructions [22].

**Figure 1:**
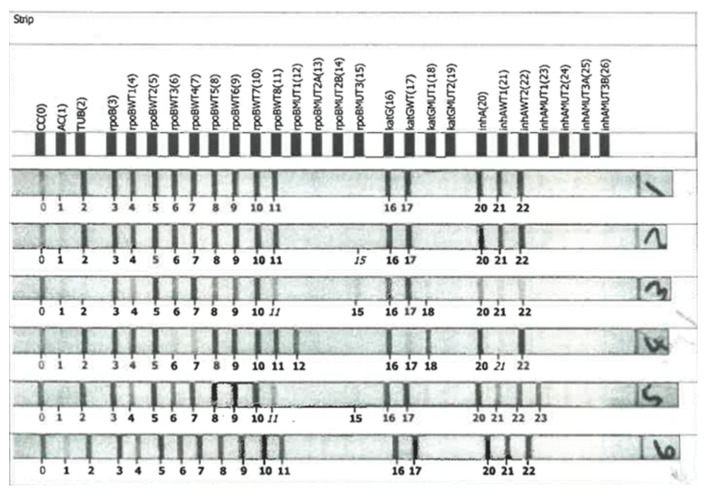
LPA strips.

The results were evaluated and interpretated by lining up conjugate control and amplification control bands with the corresponding lines on the evaluation sheet before the generated strips were adhered to in the prescribed fields. The mutation bands were deciphered using the GenoLyse package inserts. The absence of a wild-type band and the presence of a mutant band for a particular gene on the strip denoted resistance. The regions of mutations were determined by reading the mutation band location on the LPA strip (Figure 1 and Table 1).

#### 2.2.4. Spoligotyping

In total, 441 randomly chosen *M. tuberculosis* isolates were selected from the 1157 isolates, with the selection including samples that were drug monoresistant, MDR, and pre-XDR. These were genotyped using spoligotyping analysis. DNA was extracted after the samples had been heated to death. Following the manufacturer’s instructions, the fluorescence intensity was measured with a Luminex 200^®^ (Austin, TX, USA) and microbeads from the TB-SPOL Kit (Beamedex^®^, Orsay, Paris, France) to perform spoligotyping. Binary and octal formats were created from the hybridisation patterns. The generated binary codes of the isolates were added to the Pasteur Institute of Guadeloupe’s SITVIT2 database and given particular spoligotype international types (SIT) [24]. As a positive control, *M. tuberculosis* H37Rv was used to confirm quality.

## 3. Results

### 3.1. Profile of the Isolates

A total of 1157 DR-TB clinical isolates were analysed over three years: 2018, 2019, and 2020 (Table 2). Of these clinical isolates, 950 (82.1%) were drug-resistant (mono DR-TB, multidrug-resistant TB (MDR-TB), and extensively drug-resistant TB (XDR-TB)), and 207 (17.9%) were heteroresistant isolates. Table 2 shows the number (1157) of *M. tuberculosis* isolates from 2018 to 2020 and the number of heteroresistant cases among these isolates. As can be seen in Table 2, the heteroresistance rate increased over time.

### 3.2. Distribution of Mutations in the rpoB, katG, and inhA Genes

**Figure 2:**
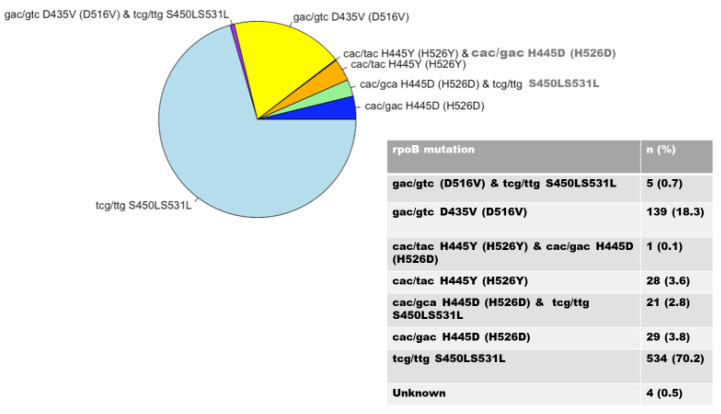
Distribution of *rpoB* gene mutations.

It was found that *rpoB 531* and *katG 315* mutations were the most prevalent regions of mutation linked to RIF and INH resistance, respectively, among 1157 *M. tuberculosis* clinical isolates. Some 761 (65.8%) of the RIF-resistant strains had known *rpoB* mutations, whereas 381 were wild-type. The *rpoB* codon S531L had the highest percentage of mutations (70.2%). Codons D516V (18.3%), H526D (3.8%), and H526Y (3.6%) had fewer mutations (Figure 2). Of the 761 isolates, 532 (69.9%) displayed mutations involving both the *rpoB* and *katG* genes, while 187 (24.6%) displayed mutations involving both the *rpoB* and *inhA* genes.

**Figure 3:**
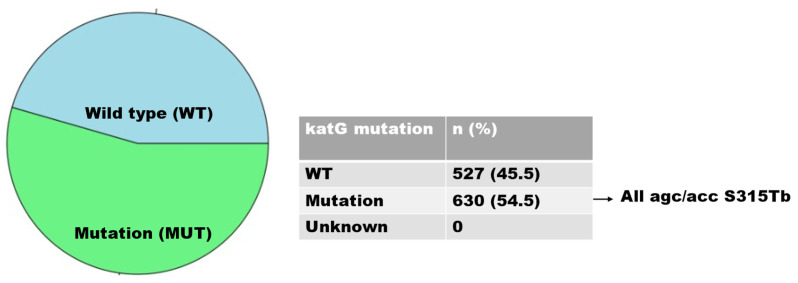
Distribution of *katG* gene mutations.

**Figure 4:**
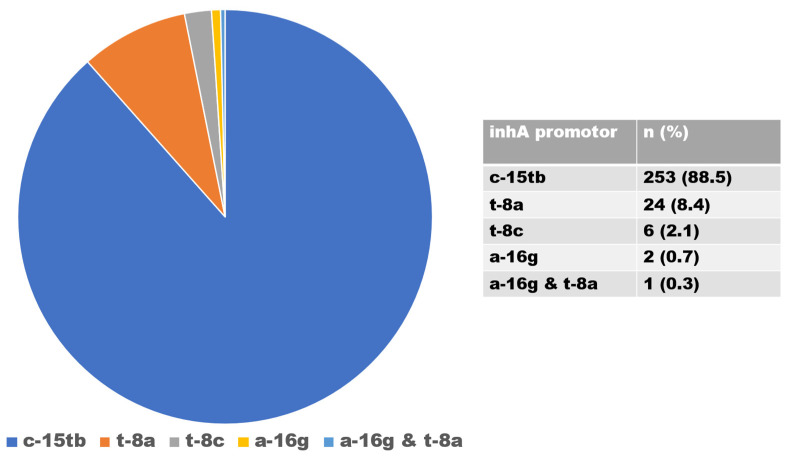
Distribution of *inhA* gene mutations.

There were 916 isolates of INH-resistant strains, of which 630 and 286 were caused by mutations in the *katG* and *inhA* genes, respectively. Mutations in the *katG* gene, associated with a high level of INH resistance, occurred in codon S315TB in 630 isolates (68.8%) (Figure 3); mutations in the *inhA* gene, associated with a low level of INH resistance, were found in 286 (31.2%) isolates and were detected in codons C-15TB (88.5%), T8A (8.4%), T-8C (2.1%), and A-16G (0.7%). Isolates with double mutations were found in codons A-16G and T8A (0.3%) (Figure 4).

Of 761 RIF-resistant strains, combined mutations involving the *rpoB* and *katG* or *rpoB* and *inhA* genes occurred in 719 samples, as displayed in Table 3 below.

### 3.3. Heteroresistant Mutations

**Figure 5:**
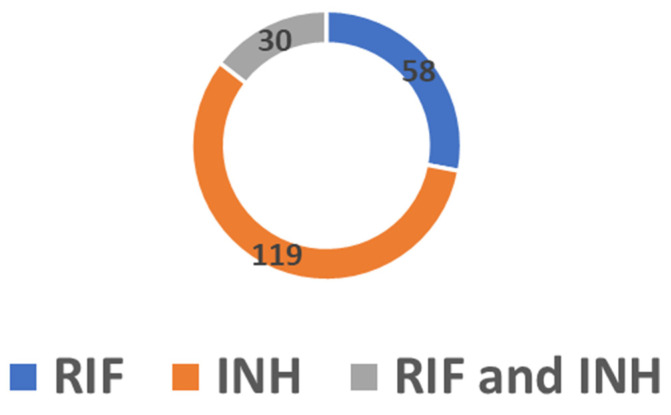
Number of cases of heteroresistance in RIF, INH and combined RIF and INH.

Of the 1157 DR-TB isolates, the overall prevalence of heteroresistance was 17.9% (n = 207). This rate increased over time, ranging from 9.6% in 2018 to 32.2% in 2020. Heteroresistance to RIF was found in 28% of cases, while heteroresistance to INH was determined in 57.5% of cases, of which 70 (58.8%) were cases of *katG*-associated heteroresistance and 49 (41.2%) were cases of *inhA*-associated heteroresistance. In addition, combined heteroresistance to RIF and INH was found in 14.5% of the DR-TB isolates (Figure 5).

### 3.4. Spoligotyping

**Figure 6:**
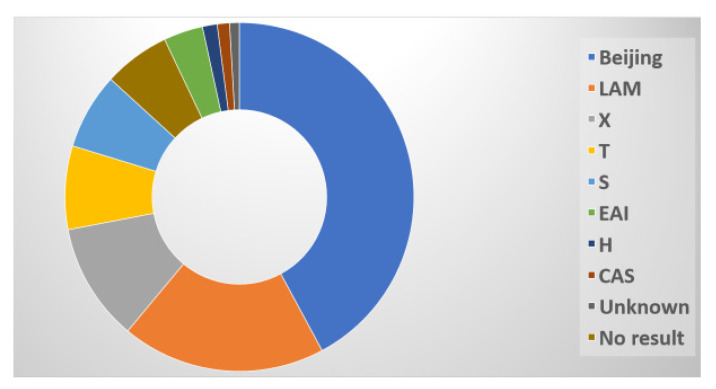
Distribution of spoligotypes.

The 441 spoligotyped isolates produced 437 unique spoligotype patterns. A pre-existing SIT in the SITVIT2 database matched the patterns of 410 isolates (93.1%), but 27 unique patterns (6.1%) were not present in the database. The Beijing family was the most prevalent genotyping lineage, with 185 (42%) isolates, followed by the LAM family with 83 isolates (18.8%), the X family with 48 isolates (10.9%), and the T family with 34 isolates (7.7%). In addition, the S family accounted for 31 isolates (7.0%), the EAI family for 16 (3.6%), the H family for 6 (1.4%), and the CAS family for 5 (1.1%) (Figure 6).

## 4. Discussion

Spontaneous chromosomal mutations in particular locations of the bacterial genome are generally thought to be the cause of resistance to RIF and INH, which several studies have proven [11]. It is uncertain how *M. tuberculosis* strains are distributed, how many have been transmitted recently (and in the past), and how DR strains transmit in the rural Eastern Cape. To our knowledge, this is the first study detailing the distribution of drug-resistant genes, mutation sites, and genotypes in the Eastern Cape.

Clinicians are concerned about MDR-TB produced by mutations in *M. tuberculosis*, which are mostly caused by the *rpoB*, *katG*, and *inhA* genes [25]. RIF resistance is typically regarded as a marker for MDR-TB. Hence, the screening of mutations in candidate genes constitutes the most significant step in making a definite diagnosis in drug-resistant strains. The 81 bp core region of the *rpoB* gene’s nucleotide sequences was examined for mutations. The prevalence of mutations in the *rpoB* gene in this study was higher than in the other genes (*katG* and *inhA* genes). Analysis of the RIF-associated mutations revealed a 65.8% prevalence of the *rpoB* gene. This is comparable to a study conducted by Otchere et al., which showed a prevalence of 52% [26]. On the other hand, the prevalence of mutations reported in other studies [11,27,28] was higher than in our investigation, at 94.9%, 93.5%, and 91.2%, respectively, all in the *katG* gene. Our analysis found that the S531L codon was the site of the majority of rifampin resistance-causing mutations in the *rpoB* gene. This finding is consistent with earlier research [11,27,29,30] and may be related to the propagation of a common clone. This codon’s mutation is known to be a hot area for *rpoB* gene mutations in *M. tuberculosis*, and it has also been observed in other South African provinces [31], which shows that these mutations are prevalent in the country. This high frequency of occurrence may be due to the low fitness cost associated with *rpoB* S531L, [31] and has been associated with major MDR-TB outbreaks. [32]. A low frequency of mutation was observed in codon 526, at 3.8% in this study, but is higher in Uganda, at 12.5% [33]; China, at 14.9%; [27] and Brazil, at 9.9% [34]; this indicates that frequency varies according to geographic location. Codons 526D and 531 co-occurred at a lower rate in this study (2.8%) than in Iran (23.9%) [35].

Owing to its early significant bactericidal activity, INH is a first-line TB medication and very important in the treatment of TB. For doctors, the detection of mutations in the *katG* or *inhA* promoter region is crucial, since the presence of these mutations predicts the degree of INH resistance and helps doctors to choose the best course of treatment [36]. Given the high degree of INH medication resistance, the catchment regions of the clinics in this study area need to be watched for any changes that may arise during patient TB treatment. In this investigation, both the *katG* S315T gene mutation and very resistant INH strains were observed. The majority of mutations occur at codon 315, being present in 30% to 90% of INH-resistant bacteria [23]. This claim was corroborated by the current study’s findings, which showed a correlation between elevated levels of INH medication resistance and S315T mutations.

Several nations, such as Zambia and Brazil, have observed a tendency for codon S315Tb mutation in the *katG* gene [11,37]. Mutations in *katG* occurred only in codon agc/acc S315Tb in this study, but Jagielski et al. [38] found that mutations occurred in eleven other codons. The prevalence of *katG* S315T varies according to the geographic region: in Sub-Saharan Africa, it is 94.9% [11]; in West Africa, it is 64% [39]; in Southeast Asia, it is 29.3% [40]; and in the United States, it is 38% [41]. The global frequency of *katG* S315 is estimated to be 64.2% [42].

In addition to the *katG* gene, the *inhA*, fabG1, and oxyR-ahpC genes are also associated with *M. tuberculosis* INH resistance. It has been discovered that 20–42% of INH-resistant bacteria carry mutations in the *inhA* promoter region [41,42]. Previous studies have shown that polymorphisms in the promoter region of the *inhA* gene cause low-level resistance to INH, which ranges from 8% to 43% [43]. In this study, the low-level resistance ratio was 31.2%, close to the high limit of the reported range. Other percentages of *inhA* mutations have been found in various parts of South Africa, including KwaZulu-Natal (27.5%) [44] and the Free State (13.4%) [10]. INH-resistant strains, comprising mutations in the *inhA* promoter region, were found in Zambia and Ethiopia at rates of 2.0% and 0.8%, respectively [11,45]. Lempens et al. [46] reported that a percentage of C-15T gene mutations, albeit low, are associated with a high level of INH resistance. This suggests that mutation of the *inhA* gene does not always indicate a low resistance level. Mutations in the *inhA* gene also give rise to cross-resistance to ethionamide (ETH), a second-line medication used in MDR therapy, and are therefore thought of as a surrogate marker for early diagnosis of ETH resistance [10,47,48]. This is because the two drugs share the same target of action. ETH was once a component of the treatment plan for MDR-TB in South Africa, according to the South African National Department of Health (NDoH). In the presence of *inhA* mutations, the use of ETH to treat MDR-TB would not have been successful due to this cross-resistance [10]. Consequently, in the clinical management of MDR-TB cases displaying *inhA* mutations, ETH must be excluded from the regimen. The majority of our C-15TB isolates (88.5%) included the most prevalent *inhA* gene mutation, supporting the findings of Seifert et al. [49], who stated that the most prevalent *inhA* gene mutation was frequently seen in C-15TB.

The clinical and molecular characteristics of *M. tuberculosis* strains vary according to geographical area. This was observed in this study, in line with the findings of Liu et al. [14]. Investigations into the evolutionary lineages of *M. tuberculosis* may benefit from knowledge of the range of mutations. The prevalence of *rpoB*, *katG*, and *inhA* mutations in various regions of Mthatha may aid in determining whether to standardize treatment plans or provide tailored care in each region in which these mutations have been discovered. There is an indication that *M. tuberculosis* strains are constantly mutating, as we observed combined mutations (Table 3). These data can be used in the development of new anti-TB drugs.

Heteroresistance in the study area increased over time. In the third year of the study period, the heteroresistance rate was almost triple that of the previous year (Table 2). The *rpoB* and *katG* combination had the highest number of heteroresistant isolates, followed by the *rpoB* and *inhA* combination. In *rpoB* and *katG* combinations, the mutation regions *rpoB* S315L and *katG* 531ST had the highest number of isolates (Table 3). Under the selective pressure of inadequate anti-TB medication, separation into susceptible and resistant organisms probably results in heteroresistance caused by infection with single strains. Several reports have documented the development of resistance as a result of insufficient treatment [50]. It has also been proven that mixed-strain infections caused by heteroresistant bacteria might have a negative effect on treatment outcomes. Treatment of such cases with first-line anti-tuberculosis medicine may select for and increase the prevalence of the drug-resistant strain in the host, because heteroresistance makes it possible for the drug-resistant strain to go undetected [51]. The heteroresistance rate of 17.9% in this study is similar to a finding by Rinder et al. [52], who reported a rate of 17%. Other studies have reported significantly lower rates [53]. In our study, one strain of either the Beijing, LAM, or X genotype induced heteroresistance, indicating that the division of a single strain into susceptible and resistant organisms is probably the main underlying mechanism.

The *M. tuberculosis* population in this study area was genetically diverse. From the 441 clinical isolates, 23 spoligotypes were observed and classified into major *M. tuberculosis* lineages: lineages 1, 2, 3, and 4, as shown in Figure 6. Beijing and Euro-American (LAM, T, S, and X) strains dominate the population structure of rifampicin-resistant tuberculosis (RRTB) isolates in South Africa, which can be explained by the historical movement of strains, as South Africa was located in a geographically central position on the historical trade route between East and West for hundreds of years [54].

One of the most widespread genotypes of *M. tuberculosis* found globally is the Beijing family, often known as lineage 2. It is usually linked to immune evasion and antibiotic resistance, which promotes rapid bacterial replication, spread, and transmission [55]. The Beijing family, which is more transmissible than other families [56], was prevalent in this study (42%). This lineage has been detected in studies reported from other parts of South Africa, including Limpopo, the Western Cape, and Mpumalanga [57,58]. According to Said et al. [58], the Beijing family, followed by LAM, are the most predominant strains in the Eastern Cape. According to a study by Chihota et al. [59], based on a review of the repository and databases, the Beijing family is the most diverse and abundant *M. tuberculosis* strain in South Africa. Furthermore, the association between HLA-B27 and host–pathogen compatibility accounts for the success of the Beijing lineage in the country [59]. Given growing concern over the prevalence of Beijing strains and their success in evolving to fit into various human groups, suitable measures should be implemented for public health surveillance. The knowledge of the lineages circulating in the study area will help us to understand the drivers of drug resistance and their impact on treatment outcomes and the management of TB transmission.

The Beijing lineage was documented for the first time in East Asian nations, according to Van Soolingen et al. [60], with a particular spoligotype pattern distinguished by the inclusion of the last ten spacers (spacers 34–43). The Beijing strains were historically introduced to South Africa, not from their primary origin (China), but from their secondary origin (Indonesia), according to historical evidence corroborated by genetic data [54]. Pokam et al. [61] argued that the dominance of the Beijing family is related to the current influx of the Asian population into Africa, as well as increased trade relations between Africa and Asia, with Africans returning from business trips to China bringing the strain with them. Various theories have been proposed regarding the introduction of the Beijing family into Africa. It is important to actively carry out surveillance of the Beijing family to verify its heightened transmission and understand its importance in the management plan of TB in this area.

In our investigation, the LAM lineage was the next most common (18.8%) after the Beijing strain. This is not unexpected, given that this genotype has been found to be widespread in the provinces of the Eastern Cape and the Free State [62,63]. This suggests that there is still continuous TB transmission throughout the province. Nonetheless, the LAM genotype predominates in Gauteng, the Northern Cape, and KwaZulu-Natal [58], and in some neighbouring countries in Southern Africa, including Zambia and Zimbabwe [11,64]. Its lowest prevalence is in the Western Cape [58]. The LAM strain is predominant in KwaZulu-Natal, which is a neighbouring province to the Eastern Cape; this proximity might eventually raise the prevalence of this strain in the Eastern Cape to the same levels as KwaZulu-Natal, exacerbating a situation in which the Beijing strain is already widespread. Hence, continuous surveillance of genetic diversity must be carried out to profile these strains.

Concerning delineation of the LAM sub-lineages, five were found in our study: LAM 3, 4, 5, 9 and LAM II-ZWE, with LAM 3 being predominant, at 74.7%. On the other hand, another study in South Africa reported that of the 12 sub-lineages delineated globally, LAM4 was the most predominant of the six predominant sub-lineages [62]. LAM11-ZWE has been reported to be the dominant sub-family in Zambia and Zimbabwe [11], with its origins traced to Portugal [59]. Two strains from the LAM 3 sub-lineage were labelled orphans, because they had no matches in the SITVIT2 database. Maguga-Phasha et al., whose study was conducted in Limpopo, found LAM 3 (7.0%) corresponding with SIT 33 only, while our investigation found LAM 3 corresponding with SIT33 and SIT 719, at 8.2% and 3.9%, respectively [57]. LAM 1, LAM 2, and LAM 6 were not reported in this study but have been reported elsewhere [37].

## 5. Conclusions

The study area has a prevalence of RIF resistance and the Beijing family of *M. tuberculosis*, both of which are associated with the development of MDR. An increase in the rate of heteroresistance was also found in this study. The identification of areas in which DR-TB is concentrated and in which TB is a burden, particularly rural areas, as in this study setting, could assist policy makers to implement targeted interventions aimed at the prevention and management of TB transmission. This is crucial in environments that have few resources, and in places with a high prevalence of DR-TB, such as the rural districts around Mthatha. Targeted interventions among rural populations are essential, since it is impossible to deliver DR-TB treatments to all groups in these locations. This is because DR-TB diagnosis and treatment are complicated by a number of factors, including co-infection with HIV and poor treatment adherence.

Since so little is known about the effects of antibiotic resistance on gene mutations, bacterial fitness and the spoligotypes of *M. tuberculosis*, it is of the utmost importance that laboratories report these, and that treating physicians have knowledge of their distribution. Continuous surveillance is advised in this study region, since genetic alterations are the cause of the high rate of resistance shown in tuberculosis treatment. The early identification of drug-resistant tuberculosis is crucial for the prevention and management of the spread of these drug-resistant variants.

## Figures and Tables

**Table 1:** Gene and mutation regions.

Gene	Band	Gene Region or Mutation
*rpoB*	MUT 1 MUT 2A MUT 2B MUT 3	D516V HS26Y H526D S531L
*katG*	MUT 1 MUT 2	S315T1 S315T2
*inhA*	MUT 1 MUT 2 MUT 3A MUT 3B	C-15T A-16G T-8C T-8A

**Table 2:** Number of DR-TB isolates over a 3-year period.

Year of Diagnosis	DR-TB Isolates *n* (%)	Heteroresistant Cases Rate *n* (%)
2018	385 (33.3)	37 (9.6)
2019	376 (32.5)	43 (11.4)
2020	396 (34.2)	127 (32.2)

**Table 3:** Isolates with predominant gene mutations and their mutation regions.

Combined Mutation	Number of Isolates (%)	Mutation Regions	Number of Isolates (%)
*rpoB* and *katG*	532 (69.9)	*rpoB* S315L and *katG* 531^ST^	366 (68.8%)
*rpoB* and *inhA*	187 (24.6)	*rpoB* S315L and *inhA* c-15tb	171 (91.4%)
		*rpoB* S315L and *inhA* a-16g	1 (0.5%)
		*rpoB* S315L and *inhA* t-8c	2 (1.1%)
		*rpoB* S315L and *inhA* t-8a	13 (6.9%)

## Data Availability

Data will be made available upon request from the corresponding author.
